# Unpacking the Medial Temporal Lobe: Separating Recollection and Familiarity

**DOI:** 10.1002/hipo.70033

**Published:** 2025-09-08

**Authors:** Andrew P. Yonelinas

**Affiliations:** ^1^ Center for Neuroscience & Center for Mind and Brain, Department of Psychology Davis California USA

## Abstract

Our understanding of how the medial temporal lobe (MTL) contributes to human cognition has advanced enormously over the past half a century. My work in the 1990s characterizing the role of recollection and familiarity processes in episodic memory led me to study the MTL's role in these two memory processes. In the current paper, I provide a personal commentary in which I describe the motivating ideas, as well as the invaluable impact of mentors, colleagues, and students that led to a series of studies showing that conscious recollection is critically dependent on the hippocampus, whereas familiarity‐based judgments are dependent on regions such as the perirhinal cortex.

## 1 Introduction

By the 1990s it had been well established that the MTL was critical for episodic memory as measured on tests of recall and recognition. This was of course based on studies of patients such as HM who became profoundly amnestic after suffering damage to the MTL (e.g., Scoville and Milner 1957). However, one thing we did not know at that time was whether amnesia reflected a selective impairment in recollection (i.e., the conscious retrieval of qualitative information about prior events) or whether it also reflected an impairment in familiarity‐based recognition (i.e., whereby recently studied items are recognized on the basis that they are perceived as more familiarity than non‐studied items). The problem was that we did not have behavioral methods that could be used to effectively separate the contribution of these processes to overall memory performance. Some early work had suggested that amnesic patients were particularly impaired at discriminating between recently and frequently presented items, and this was interpreted as suggesting that they were severely impaired at recollecting episodic details (e.g., Huppert and Piercy 1976). In addition, amnesic patients were often more impaired on tests of free recall, which presumably relied heavily on recollection, than they were on tests of recognition which were thought to rely more on familiarity (e.g., Aggleton and Shaw 1996). However, one concern was that some memory tests may just be more demanding and so may be more sensitive to disruption. In addition, recognition memory was generally impaired in these patients, and so it was unknown whether familiarity was preserved or whether it too was disrupted.

The second major gap in our understanding was whether the memory impairments observed in amnesia reflected damage to the hippocampus per se or to other cortical regions within the MTL because the patients that had been studied often suffered widespread damage that included the hippocampus and the surrounding MTL tissue. To answer this question, we needed to test patients with selective lesions either to the hippocampus or to the surrounding MTL tissue.

## 2 Developing Methods to Measure Recollection and Familiarity

The road that led me to become involved in addressing these questions was rather indirect, but it began in the early 1990s when I was interviewing for graduate school and met Larry Jacoby for the first time. The lasting impression I had from the meeting was that Larry had an absolutely infectious passion for science. During the interview, he described a new approach he was developing to mathematically separate the effects of recollection and familiarity on overall memory performance, that involved what seemed to me like an impossibly complicated set of equations. Even though I had only a fuzzy idea of what those equations meant, it seemed like Larry was on to something important, and I felt I had no choice but to go along for the ride. The critical insight behind Larry's approach was to include not just studied items and new items in the test list, as was common in tests of recognition memory in those days, but to also include items that were similar to the studied items (e.g., items that may have been studied in a different context or they may have been modified in some way such as having been studied in a red color rather than in green) (Jacoby 1991). At test, subjects would be instructed to respond “yes” to studied items and respond “no” to both the new items and the similar lures. Recollection was then measured as the probability that subjects could discriminate between studied items and similar lures, and familiarity was then estimated as the propensity to incorrectly recognize a similar lure given that it was not recollected.

During my time in Larry's lab, we tested several variations of his “process dissociation procedure” (i.e., PDP), conducted studies to address potential critiques of the method, and used the method to examine the functional nature of recollection and familiarity. For example, we found that these processes were functionally dissociable in the sense that they were differentially sensitive to factors like response speeding, attention, interference and perceptual similarity. These functional dissociations showed that recollection and familiarity reflected distinct memory processes, rather than simply reflecting strong compared to weak memories, and so this led us to wonder whether these processes might also be neuroanatomically distinct.

One of the many lessons I learned during my time in Larry's lab was that it is never a good idea to rely on a single experimental method because the underlying assumptions of any method can be violated, and for any single method, there will always be multiple alternative interpretations (as many of our colleagues at the time were happy to point out. e.g., Curran and Hintzman 1995). Science, as Larry described it, demanded that one observe convergence from across a number of different methods. With this in mind, we developed a set of measurement procedures that used entirely different methods and analytical approaches. For example, whereas the PDP measured recollection and familiarity using objective measures of discriminability such as the ability to accurately remember when or where events took place, we developed an approach inspired by Endel Tulving's remember/know procedure (Tulving 1985) in which these processes were measured based on asking subjects to introspect and report whether each recognition response was accompanied by the conscious recollection of specific details about a study episode or on the basis that it was familiarity in the absence of recollection. Although subjective report methods made a number of our behaviorist colleagues rather uncomfortable, it seemed to us that ignoring this critical aspect of human memory would be a mistake. In fact, we found that the subjective reports converged remarkably well with the objective PDP estimates of recollection and familiarity, indicating that subjects had conscious access to these underlying processes. In addition, we developed signal‐detection‐based analyses of confidence responses (i.e., Receiver Operating Characteristics (ROCs), See Hautus et al. 2021; Yonelinas 1994) that allowed us to systematically separate effects of these memory processes from the effects of response bias. The basic idea behind this method is that if a subject can recollect specific information about a study event in which the item was encoded (e.g., I can remember that it was the 2nd item in the study list) they should be confident that it was studied. In contrast, items that are familiar but not recollected should be less confidently recognized (at least on average). Thus, by examining recognition confidence responses, one can derive estimates of recollection and familiarity. Although we did not apply these methods to study the effects of amnesia, that early work established the methods that would ultimately prove quite useful.

## 3 Assessing the Role of the MTL in Recollection and Familiarity

In 1995 I accepted a faculty position at the University of California Davis and was lucky enough to meet Bob Knight and Neal Kroll, who both had experience testing human amnestic patients and were willing to educate a young cognitive researcher in the complexities of neuropsychological research. Working with Neal's graduate student Ian Dobbins and Michelle Lazzara, who was one of my first graduate students, we began to explore the role of the MTL in episodic memory. At the time, I imagined that we could solve the puzzle with one or two simple experiments and was sure of exactly what we would find. Of course, I was entirely wrong. My expectation was that amnesic patients with MTL damage would be impaired in recollection but not familiarity. This prediction was based on prior work showing that implicit forms of memory were entirely preserved in amnesics—even those with extensive MTL damage—and a growing body of research indicating that familiarity exhibited functional characteristics very much like those exhibited by implicit forms of memory.

In our first study, we applied various measurement methods to examine recollection and familiarity in patients with extensive MTL damage (Yonelinas et al. 1998). To my surprise, we found that patients with extensive damage that included both the hippocampus and the surrounding MTL exhibited deficits in both recollection and familiarity. These effects were observed in process dissociation, remember/know, and ROC studies, and the results clearly ruled against the possibility that the MTL was critical for recollection but not for familiarity. However, it did not rule out other plausible accounts. For example, one influential theory at the time assumed that the MTL operated as a unified declarative system and so should equally support both recollection and familiarity (Squire 1992). Although the recollection deficits were more pronounced than the familiarity deficits, we could not rule out this single‐system view. In contrast, however, Howard Eichenbaum, Tim Otto, and Neal Cohen (Eichenbaum et al. 1994) argued that results from rodent memory studies suggested that the hippocampus was critical for long‐term “relational memory,” which seemed quite similar to recollection, whereas the surrounding parahippocampal region supported a more temporary form of memory for individual items, which seemed more similar to familiarity. In addition, independent work based largely on rodent studies by Aggleton and Brown (1999) suggested that a hippocampal–anterior thalamic axis was critical for recollection, whereas a perirhinal–medial dorsal thalamic axis was critical for familiarity. However, we did not know whether any of these proposals could account for the deficits observed in human amnesic patients.

Our next step was to examine memory in mild hypoxic patients who had suffered from cardiac arrest and were expected to exhibit relatively selective hippocampal lesions that had limited effects on surrounding MTL regions. Across a variety of measurement paradigms, we found that the hypoxic patients exhibited selective recollection impairments, whereas a different group of patients who had damage that included both the hippocampus and the surrounding MTL cortex exhibited significant deficits in both recollection and familiarity (Yonelinas et al. 2002). The results indicated that recollection and familiarity were neuroanatomically distinct, but one limitation of that initial study was that the hypoxic patients could not be scanned because they had pacemakers, and so we could not verify that their lesions were entirely restricted to the hippocampus. Subsequent studies, however, confirmed that selective hippocampal lesions (Aggleton et al. 2005) as well as selective fornix damage (Rudebeck et al. 2009) were sufficient to lead to a selective recollection impairment, verifying that the hippocampus was critical for recollection but not for familiarity.

A limitation of human lesion studies is that it is always possible that the brain damage may extend beyond the regions of interest (e.g., neuroimaging and histological examinations may fail to detect subtle damage outside of the hippocampus). One way of addressing this issue is to use rodent studies in which one has considerably more control over lesion location. Howard Eichenbaum and researchers in his group such as Norbert Fortin, Sean Wright, Cullen Owens, and Magdelana Sauvage had been interested in examining recollection and familiarity in rodents and suggested to me that the ROC methods we had been using in human amnesic studies might also work in rodents (Fortin et al. 2004; Sauvage et al. 2008). I will admit I was initially skeptical that we could convince rats to vary their response criteria in ways that would allow us to assess ROCs, but Norbert enthusiastically took on the task. After a few months I received a rather cryptic email from Howard with a single figure that contained two ROCs. I immediately recognized them as the ROC results from our 2002 study in which we found that compared to healthy controls, hypoxic patients had selective deficits in recollection. I asked Howard why he had sent me a copy of my earlier amnesia results. He quickly responded that he had not accidentally sent me a copy of my old human data but rather he had sent the ROCs from the study they had just completed. The fact that the amnesic and rodent lesion data converged so precisely was very reassuring, but perhaps an even more remarkable take‐away from this work was that these complex memory processes that many people at the time thought were so uniquely human also appeared so clearly in rodents.

Studies of nonhuman primates have also begun to explore the role of recollection and familiarity in recognition. For example, an examination of recognition in nonhuman primates has led to results that are similar to those seen in humans, indicating that overall performance reflects both recollection and familiarity (Guderian et al. 2011; Basile et al. 2023). However, whether hippocampal lesions in nonhuman primates selectively disrupt recollection as they do in rats and humans has not yet been tested.

We then wondered whether the opposite dissociation might also be observed (i.e., would selective damage to the surrounding MTL cortex such as the perirhinal cortex lead to selective deficits in familiarity?), but we were not aware of any patient with such a lesion. Fortunately, Stefan Köhler had identified a patient (i.e., patient NB) who had a surgical resection of the left perirhinal cortex for epilepsy that did not impact the hippocampus. So along with Ben Bowles, who was a graduate student in Stefan's lab, we were able to test this individual using the measurement methods my lab had developed, along with a new response deadline procedure that Stefan and Ben had developed (Bowles et al. 2007). The response deadline procedure leveraged the fact that familiarity is found to become available faster than recollection, and thus performance should rely more on familiarity when subjects are forced to respond very quickly at test (for the use of similar approaches to studies of nonhuman primates see Basile and Hampton 2013; Wu and Buckley 2022). The results from each of those experiments indicated that damage to the perirhinal cortex could in fact lead to a selective familiarity deficit, and subsequent patient studies have since verified the results (Brandt et al. 2016; Argyropoulos et al. 2022).

Another worrisome limitation of lesion studies is that lesioned brains can reorganize, and this can obscure attempts to localize brain functions. This fact is something that my colleague Charan Ranganath is particularly fond of reminding me about, and this has led us to a very productive and fun series of collaborative neuroimaging studies over the years. There have been more than a few “beer bets” regarding exactly what patterns of results we would observe (I won a few and lost a few more). However, one finding that we were both quite happy to see that converged quite nicely with the lesion results was that hippocampal activity during memory encoding predicted subjects' ability to recollect details of the study event but was not predictive of familiarity‐based responses, whereas activity in the perirhinal cortex was related to familiarity but not to whether subjects recollected study details (e.g., Ranganath et al. 2004). So even in healthy brains, the hippocampus and perirhinal cortex are involved in supporting recollection and familiarity, respectively.

The period between 1990 and 2010 provided important insights into the role of the MTL in episodic memory, but of course more recent work has continued to provide additional insights and has indicated that the story of the hippocampus is much more interesting and nuanced than we had originally thought. For example, we now know these regions do not operate in isolation but rather involve complex interactions among a broad network of brain regions (Ranganath and Ritchey 2012; Rugg and Vilberg 2013). Moreover, we know that there is no simple one‐to‐one mapping between these processes and single brain regions—the hippocampus, perirhinal cortex, and parahippocampal cortex support distinct roles and act together to contribute to recollection and familiarity (Eichenbaum et al. 2007). Moreover, these different MTL regions play roles in many other cognitive functions far beyond supporting episodic memory, including conceptual implicit memory (Wang et al. 2010), working memory (e.g., Goodrich and Yonelinas 2016), and even in visual and auditory perception (Aly et al. 2013; Graham et al. 2010; Hawkins et al. 2025; Murray et al. 2007). Finally, important advances have been made characterizing the functional heterogeneity within these MTL regions (e.g., Kafkas et al. 2017).

So, were there any major discoveries that we made during this period that I can lay claim to? I do not think so, but I do know that thanks to the collaborative efforts of my many colleagues, together we have begun to unpack the role of the hippocampus in cognition.

## Conflicts of Interest

The author declares no conflicts of interest.

## Data Availability

Data sharing not applicable to this article as no datasets were generated or analyzed during the current study.
